# RhoA/ROCK pathway mediates the effect of oestrogen on regulating epithelial‐mesenchymal transition and proliferation in endometriosis

**DOI:** 10.1111/jcmm.15689

**Published:** 2020-07-29

**Authors:** Zhi‐Xiong Huang, Xiao‐Mei Mao, Rong‐Feng Wu, Shao‐Min Huang, Xin‐Yu Ding, Qiong‐Hua Chen, Qing‐Xi Chen

**Affiliations:** ^1^ School of Life Sciences Xiamen University Xiamen China; ^2^ Reproductive Medical Center The First Affiliated Hospital of Xiamen University Xiamen China; ^3^ The Key Laboratory of Research and Diagnosis of Gynecological Diseases of Xiamen City and Department of Obstetrics and Gynecology The First Affiliated Hospital of Xiamen University Xiamen China

**Keywords:** endometriosis, epithelial‐mesenchymal transition, ERK, oestrogen, oestrogen receptor α, proliferation, RhoA/ROCK pathway

## Abstract

Endometriosis is a benign gynaecological disease appearing with pelvic pain, rising dysmenorrhoea and infertility seriously impacting on 10% of reproductive‐age females. This research attempts to demonstrate the function and molecular mechanism of RhoA/ROCK pathway on epithelial‐mesenchymal transition (EMT) and proliferation in endometriosis. The expression of Rho family was abnormally changed in endometriotic lesions; in particular, RhoA and ROCK1/2 were significantly elevated. Overexpression of RhoA in human eutopic endometrial epithelial cells (eutopic EECs) enhanced the cell mobility, epithelial‐mesenchymal transition (EMT) and proliferation, and RhoA knockdown exhibited the opposite function. Oestrogen up‐regulated the RhoA activity and expression of RhoA and ROCK1/2. RhoA overexpression reinforced the effect of oestrogen on promoting EMT and proliferation, and RhoA knockdown impaired the effect of oestrogen. oestrogen receptor α (ERα) was involved with the regulation of oestrogen on EMT and proliferation and up‐regulated RhoA activity and expression of RhoA and ROCK1/2. The function of ERα was modulated by the change in RhoA expression. Furthermore, phosphorylated ERK that was enhanced by oestrogen and ERα promoted the protein expression of RhoA/ROCK pathway. Endometriosis mouse model revealed that oestrogen enhanced the size and weight of endometriotic lesions. The expression of RhoA and phosphorylated ERK in mouse endometriotic lesions was significantly elevated by oestrogen. We conclude that abnormal activated RhoA/ROCK pathway in endometriosis is responsible for the function of oestrogen/ERα/ERK signalling, which promoted EMT and proliferation and resulted in the development of endometriosis.

## 1 INTRODUCTION

Endometriosis is a complex gynaecological disease, which is identified as the presence of endometrium‐like tissues outside the uterine cavity and impacts 6%‐10% female of reproductive age.^1^ Endometriosis is considered as an oestrogen‐dependent and inflammatory disorder that is usually accompanied by dysmenorrhoea, chronic pelvic pain, dyspareunia and infertility.^2^, ^3^, ^4^ Furthermore, the recurrence rate of endometriosis is pretty high that severely influences patients’ health and quality of life.^5^, ^6^

Oestrogen fuels the cell proliferation, promotes inflammation and inhibits apoptosis, and it is regarded as one of the factors that promotes the development of endometriosis.^7^, ^8^, ^9^ Studies found that aromatase and oestrogen kept high level in endometriosis tissues and survived the cells in ectopic locations.^10^, ^11^ Furthermore, other studies have reported the aberrant level of EMT is present in endometriosis and oestrogen induces cell proliferation, invasion, migration and EMT‐related markers in both normal and endometriotic epithelial cells.^12^ Zhang et al found that oestradiol promoted EMT via MALAT1/miR200s sponge function.^13^ Therefore, the development of endometriosis is affected by oestrogen in many ways and numerous signalling pathways are involved in the process, but the in‐depth mechanism deserves further exploration. On the other hand, it was reported that uterine receptivity defects were triggered by ERα down‐regulation in the mid‐secretory phase.^14^ Consequently, ERα is extraordinary significant for the endometrium to perform normal physiological functions. Other study found that the polymorphisms of ERα are useful markers for predicting endometriosis susceptibility.^15^ But there is not adequately revealed relationship and mechanism of ERα and oestrogen in development in endometriosis.

Rho family serve as molecular switches involved in the regulation of diverse cellular functions including cytoskeletal organization, growth and differentiation.^16^ It is reported that RhoA, RhoC and ROCK1 were significantly higher in ectopic endometrial cells compared with the eutopic endometrial cells in endometriosis, which involved with promoted migration of endometrial cells of endometriosis.^17^ Yuge et al claimed that the increased expression of RhoA, ROCK1 and ROCK2 proteins of endometriotic stromal cells may be involved in the pathogenesis of endometriosis‐associated fibrosis.^18^ Other report considered the increased RhoC expression detected in endometriotic lesions might be among the key elements involved in the origin and the maintenance of endometriosis.^19^ However, all of these researches lack comprehensive cognition and mechanism exploration about the Rho family in endometriosis, and the factors affecting the function of Rho family are little known. Further study on the role and mechanism of Rho family in endometriosis may have profound significance as a treatment for endometriosis.

In the present study, we aimed to investigate the expression of Rho family and its function on EMT and proliferation in endometriosis. Further researches were employed to assess the association between Rho family and oestrogen pathway and the molecular mechanism. We also sought to examine whether all the results presented in endometriotic mouse model.

## 2 MATERIALS AND METHODS

### 2.1 Reagents

Collagenase Ⅳ (#A004186‐0001; Sangon Biotech; Shanghai, China) and DNase I (#B002004‐0005; Sangon Biotech) were employed to isolate primary human eutopic EECs. DMEM/F12 (1:1) medium (#SH30023.01; HyClone, Shanghai, China) and foetal bovine serum (FBS; #04‐001‐1A; Biological Industries, Cromwell, USA) were used to culture eutopic EECs. 17β‐Oestradiol/oestrogen/E2 (#E2758) and PD98059 (#P215) were got from Sigma‐Aldrich (Shanghai, China). GAPDH mouse monoclonal antibody (#40493) was procured from ABclonal (Boston, USA). RhoA (#ab187027), ROCK1 (#ab219587) and ROCK2 (#ab71598) antibodies were obtained from Abcam (Cambridge, UK). E‐cadherin (#14472), Vimentin (#5741), N‐cadherin (#13116), phosphorylated ERK1/2 (#4370) and total ERK1/2 (#4695) antibodies were from Cell Signaling Technology (CST; Danvers, MA, USA).

### 2.2 Patients and primary cell culture

A 20 normal female patients and 26 patients suffering from ovarian endometriotic cyst surgery were recruited from Department of Obstetrics and Gynecology in the First Affiliated Hospital of Xiamen University (Table 1). The normal patients without history of endometriosis were confirmed by ultrasonography. All patients had regular menstrual cycles and were without hormone treatment for more 3 months before the surgery. The endometriotic lesions were collected after surgery and examined by pathology. The use of samples gated permission from the ethics committee of hospital, and all patients signed the informed consent.

**TABLE 1:** Clinical information of normal and endometriosis patients

Parameters	Normal group	Endometriosis group
No. of case	20	26
Age, years	33.16 ± 6.48	34.00 ± 4.54
CA125	34.03 (18.75‐66.63)	28.59 (20.32‐137.00)
CA129	43.40 (27.08‐98.58)	51.6 (36.13‐85.88)
ARSM stage^a^		
I	—	5
II	—	9
III	—	8
Ⅳ	—	4

^a^ Revised American Society for Reproductive Medicine Classification (rASRM: American Society for Reproductive Medicine, 1997).

The separation of primary cells from eutopic endometrial tissues was executed as previous report.^20^ Briefly, the endometrial tissues were cut into small pieces and then dissociated by Collagenase Ⅳ and deoxyribonuclease for an hour. Then, the tissue suspension was filtered by nylon cell strainers to separate epithelial and stromal cells. The separated cells were cultured within DMEM/F12 containing 10% FBS in dishes in 10% CO_2_ incubator at 37℃.

### 2.3 Drug treatment

The separated human eutopic EECs were digested by trypsin and cultured within DMEM/F12 medium without FBS for 24 hours to remove endogenous steroids. 100 nmol/L oestrogen or 10 μmol/L PD98059 (inhibitor of ERK) were used to treat cells for 48 hours to analyse the effect on EMT and proliferation. To evaluate the time‐dependent character of ERK phosphorylation upon oestrogen, the eutopic EECs were treated with 100 nmol/L oestrogen for 0, 1, 5, 10, 15 and 30 minutes. The eutopic EECs disposed to medium containing 0.01% DMSO were applied as vehicle controls.

### 2.4 Database source

The gene expression of Rho/ROCK pathway was analysed referred to the GEO database (https://www.ncbi.nlm.nih.gov/geo/). GSE25628 was selected as target gene expression profile, which was based on the GPL571 platform ([HG‐U133A_2] Affymetrix Human Genome U133A 2.0 Array). All the data were freely available online, and this study did not involve any experiment on humans or animals performed with any of the authors.

### 2.5 Establishment of gene‐modified cell

Human RhoA or ERα coding regions were respectively cloned and inserted into GV208 vector (GeneChem, Shanghai, China) to generate the overexpressed vector plasmid. The small interfering RNA (siRNA) was used to suppress RhoA or ERα, and the designed siRNA fragments were inserted into GV112 to obtain the knockdown vector plasmid. 293T cells were transfected with overexpressed or knockdown vector plasmid and lentivirus packing plasmids (VSVG/PMDL/REV) for 48 hours to generate lentivirus. The human eutopic EECs were cocultured with viral supernatants for 24 hours to generate cells with modification of RhoA or ERα expression.

### 2.6 Quantitative real‐time polymerase chain reaction (qRT‐PCR)

RNAiso Plus (#9108; Takara Biotechnology; Kyoto, Japan) was used to lyse cells or tissues. And then, the mRNA was extracted by chloroform, precipitated by isopropyl‐ketone and purified by 75% ethanol. cDNA was synthesized from mRNA via using the PrimeScript RT Reagent Kit (#9108; Takara Biotechnology; Kyoto, Japan). qRT‐PCR was performed with the TB Green Premix Ex Taq II (#RR820A, Takara) in the LightCycler 480 system (Roche Molecular Biochemicals, Mannheim, Germany). The results were normalized based on GAPDH mRNA level. The primer sequences used in qRT‐PCR are listed in Table S1.

### 2.7 Western blot

The protein samples from eutopic EECs or tissues were exacted by RIPA lysis buffer (#ab156034; Abcam, Cambridge, UK) with protease inhibitors and phosphatase inhibitor. The preparation of these proteins was by SDS‐PAGE loading buffer. And then, the proteins were separated by 10% SDS‐PAGE and transferred onto PVDF membranes (#03010040001; Roche, Basel, Switzerland). Different primary antibody dilutions and corresponding HRP‐labelled secondary antibody were used to incubate the membranes. The dilution rates of antibody in the experiment were E‐cadherin (1:500), vimentin (1:1000), N‐cadherin (1:1000), RhoA (1:500), ROCK1 (1:5000), ROCK2 (1:20 000), total ERK (1:1000), phosphorylated ERK (1:1000), GAPDH (1:10 000) and secondary antibody (1:10 000). The signal was finally visualized by an enhanced chemiluminescence (ECL). Blots were scanned and analysed using ChemiDoc MP Imaging System (Bio‐Rad; California, USA). ImageJ software (1.52u version) was used to calculate to greyscale value of immunoblot.

### 2.8 RhoA activation assay

RhoA activation assays kit (STA‐403A; Cell Biolabs; San Diego, USA) were performed to quantify the activity of RhoA. After the appropriate treatment, eutopic EECs were lysed by lysis buffer in ice bath. The lysate was collected and centrifuged to obtain the supernatant. The Rho‐binding domain (RBD) beads were incubated within the supernatant for 1 hour in ice bath. Then, the mixture was centrifuged and the supernatant was abandoned. The beads were washed by 1× Assay Buffer for 3 times and resuspend in 40 µL of 2× reducing SDS‐PAGE sample buffer. The prepared samples were then performed with SDS‐PAGE and electronically transferred onto PVDF as described in Western blot. The detection of activated RhoA (GTP‐RhoA) was determined by usage of anti‐RhoA antibody.

### 2.9 Transwell assays

To evaluate the function of RhoA, transwell assays were performed to detect the ability of cell migration. 1 × 10^5^ RhoAoe or siRhoA eutopic EECs were suspended in DMEM/F12 containing 0.1% bovine serum albumin (BSA) and planted into 8‐μm pore size transwell chambers (#3422; Corning; NY, USA). The bottom wells were added 500 μL DMEM/F12 containing 10% FBS. After 24 hours, the lower surface of the chambers was fixed by 75% ethanol and stained by crystal violet, and the migrated cells were counted and captured randomly by microscope (Olympus Corporation, Tokyo, Japan).

### 2.10 Cell proliferation assay

To investigate the proliferation of eutopic EECs suffering from different treatment, Cell Counting Kit‐8 (CCK‐8; #HY‐K0301; MedChemExpress, Shanghai, China) was employed to quantify the growth rate. 1 × 10^4^ eutopic EECs were seeded into 96‐well plates within DMEM/F12 medium without FBS for 24 hours to prevent the influence of endogenous oestrogen. And then, the cell culture was changed to 100 μL fresh medium with drugs. After treating with for 48 hours, 10 μL CCK‐8 was added into each well and reacted for 2 hours at 37°C. The absorbance of supernatants was measured with an ELISA reader spectrophotometer (Dynatech Laboratories, Chantilly, VA, USA).

### 2.11 EdU labelling assays

EdU labelling assays were performed as described in instructions of EdU assay kit (#C0071S; Beyotime Biotechnology, Shanghai, China). 1 × 10^4^ eutopic EECs were planked in 24‐well plates and treated with drugs or transfection by lentivirus for 48 hours. After treatment with EdU solution for 3 hours, the cells were fixed by 4% paraformaldehyde and permeabilized by PBS containing 0.3% Triton X‐100. And then, the click additive solution (include in the kit) was added to incubate the cells for 30 minutes in the dark conditions. Finally, Hoechst 33342 was used to stain the nucleus. The results were observed and captured using fluorescence microscope (Olympus Corporation, Tokyo, Japan).

### 2.12 Immunochemistry (IHC)

The human and mouse tissue samples were fixed by formalin solution and embedded by wax. These tissue waxes were cut into slides, which were then routinely dewaxed, dehydrated and antigen‐repaired using pressure cooker boiling by 10 mmol/L sodium citrate buffer. The slides were incubated with the primary antibodies (RhoA, 1:100; ROCK1, 1:100; and ROCK2, 1:100; phosphorylated ERK, 1:100) at 4°C overnight. After washing by PBS for 3 times, the slides were incubated with corresponding secondary antibodies (1:100). Finally, DAB Color Development Kit (#AR1022; Boster Biological Technology, Wuhan, China) was used to stain the slides. The results were observed and captured by fluorescence microscope (Olympus Corporation, Tokyo, Japan). HE staining was similar to immunochemistry but without usage of antibodies.

### 2.13 Endometriotic Mouse Model assay

The endometriotic mouse model was established referred to the previously described method with minor modifications.^21^, ^22^ Female BALB/c mice (6‐8 weeks old and 15‐20 g) were purchased and raised in Animal Research Laboratory of Xiamen University. The operations on mice were in accordance with animal ethics. All the mice were ovariectomized to eliminate effect of endogenous oestrogen. The mice were divided into donor group (10 mice), control group (Ctrl group, 10 mice) endometriotic groups (EM group, 10 mice) and oestrogen‐treated endometriotic groups (E2/EM group, 10 mice).

The donor mice were killed, and the uterine horns and myometrium were peeled off to obtain the endometrium‐rich fragments. And then, the endometria were cut into pieces and equally injected into the abdominal cavity of EM and E2/EM group mice. The mice of E2/EM group were abdominal injected 1mL PBS containing oestrogen (0.6 g/L), and EM group was injected with 1 mL PBS every day. The mice were monitored for endometriotic lesion growth for one month, and uterine and ectopic lesions were collected, respectively.

### 2.14 Statistical analysis

All data were analysed by GraphPad Prism 7 software (San Diego, CA, USA). Comparison of data was performed with Student's t test or one‐way analysis of variance (ANOVA). *P* value < 0.05 was deemed to be statistically significant.

## 3 RESULTS

### 3.1 The expression of RhoA and ROCK1/2 was significantly increased in endometriosis

**FIGURE 1:**
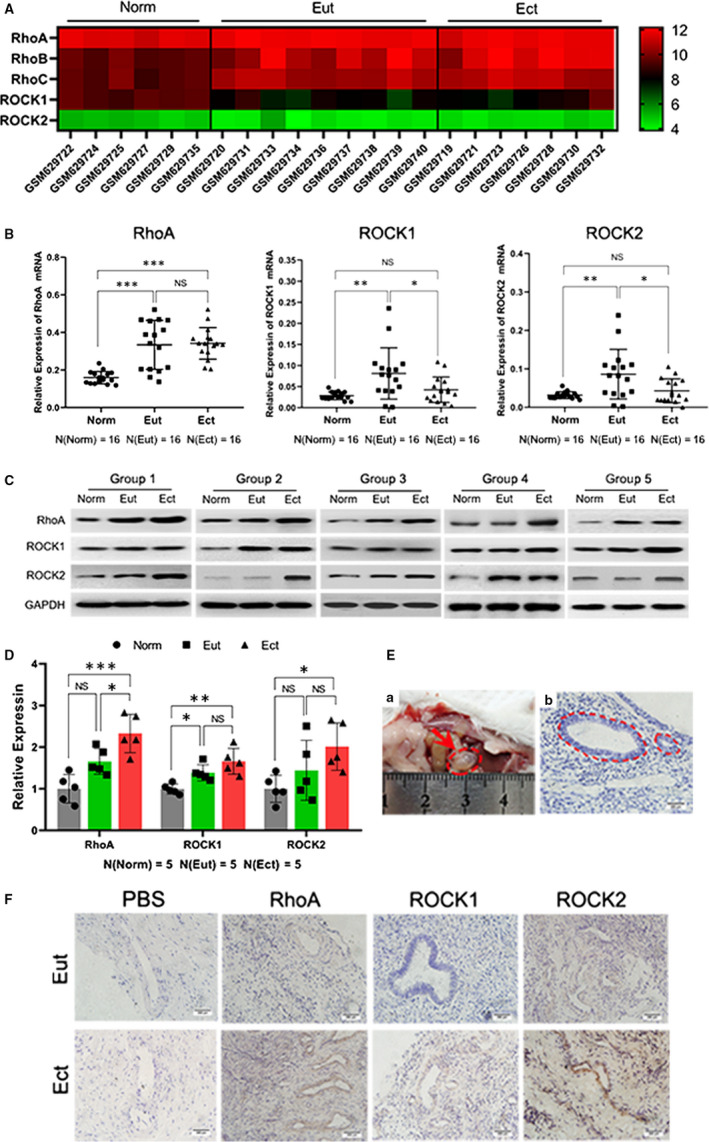
The expression of RhoA/ROCK pathway was significantly increased in both human and mouse endometriotic lesions. A, The expression of Rho family including RhoA, RhoB, RhoC, ROCK1 and ROCK2 referring to GEO database. Norm: eutopic endometria from normal patients, Eut: eutopic endometria from endometriosis patients, Ect: ectopic endometriotic lesions B, The mRNA expression of RhoA (left), ROCK1 (middle) and ROCK2 (right) analysed by qRT‐PCR. C, The protein level of RhoA, ROCK1 and ROCK2 via Western blot assays in 5 groups including normal endometria, eutopic endometria and ectopic lesions. D, Quantitative analysis of RhoA, ROCK1 and ROCK2 proteins. E, Establishment of endometriotic mouse model (right) and characterized by HE staining (left). F, The expression of RhoA, ROCK1 and ROCK2 detected by IHC in eutopic endometria and ectopic lesions from mouse model of endometriosis after treating for 1 mo. Data are expressed as mean ± SEM. NS = no significant difference, *P < 0.05, **P < 0.001, ***P < 0.0001 vs normal group

To explore the expression of Rho/ROCK pathway in endometriosis, GEO database was queried to assess the expression of RhoA, RhoB, RhoC, ROCK1 and ROCK2. As shown in Figure 1A and Figure S1A, all the protein expression of Rho/ROCK pathway was abnormally changed. To confirm these results, qRT‐PCR was employed to qualify the mRNA level of Rho/ROCK pathway. As in Figure 1B and Figure S1B, all the mRNA of Rho/ROCK pathway was abnormally expressed in eutopic endometria and ectopic lesions compared with normal endometria. Among these results, the level of RhoA mRNA was significantly enhanced compared with RhoB and RhoC. Thus, further investigations were performed to explore the proteins level of RhoA and ROCK1/2. The results of Figure 1C,D suggested that RhoA, ROCK1 and ROCK2 proteins were significantly elevated in eutopic endometria and ectopic lesions and the ectopic endometria presented the most dramatic increasement. To assess RhoA/ROCK pathway in vivo, endometriosis mouse model was established and characterized (Figure 1E). And RhoA, ROCK1 and ROCK2 were remarkably increased in the endometriotic lesions of mice (Figure 1F). Thus, we concluded that RhoA/ROCK pathway was intensively promoted in endometriosis.

### 3.2 RhoA modulated the EMT and proliferation of eutopic EECs

**FIGURE 2:**
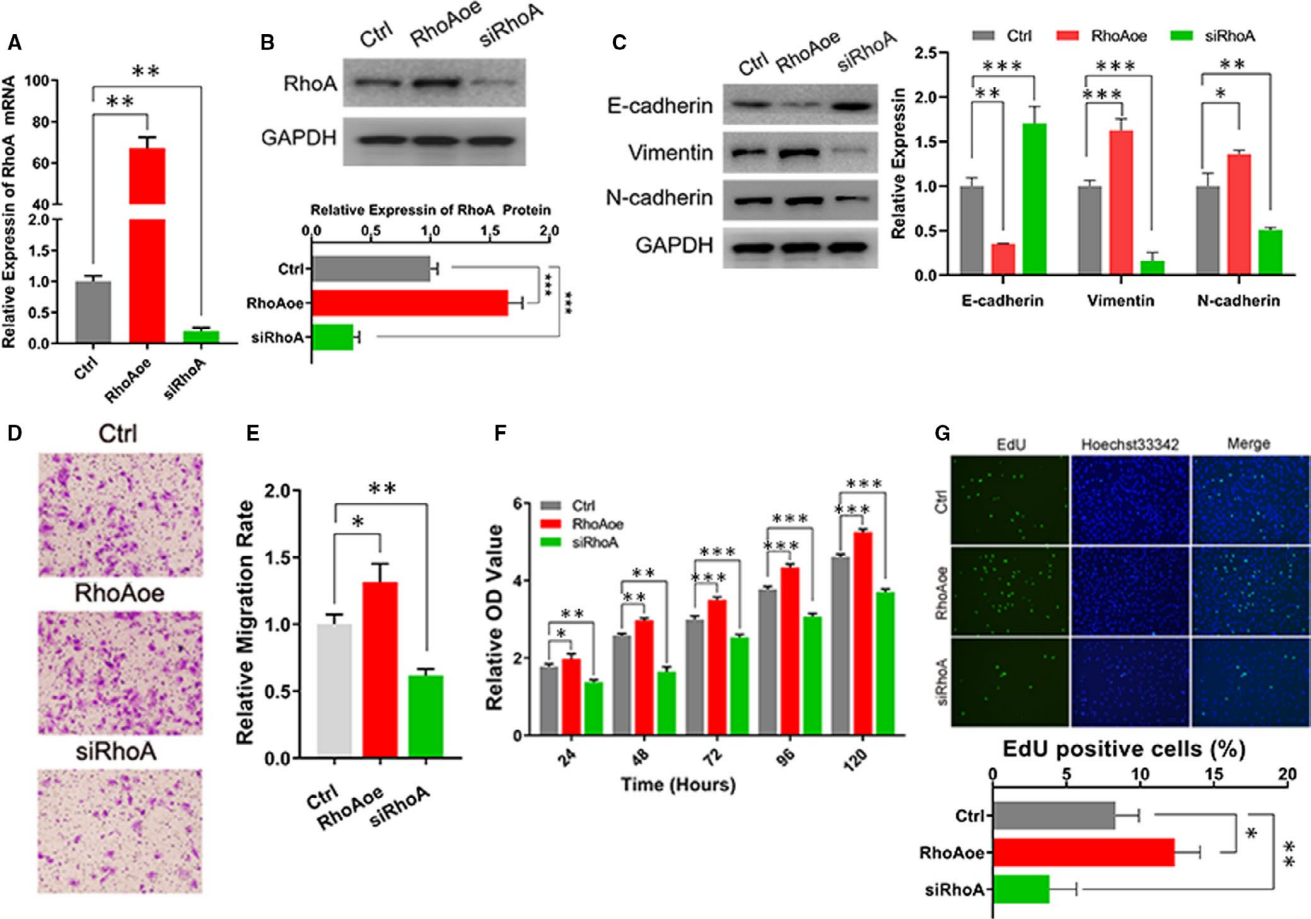
The cell mobility, EMT and proliferation were promoted by RhoA. A, The expression of RhoA assessed in human eutopic EECs, RhoA‐overexpression EECs and RhoA‐knockdown EECs by RT‐qPCR. Ctrl: control eutopic EECs, RhoAoe: RhoA‐overexpressed cells, siRhoA: RhoA‐knockdown cells. B, The protein level of RhoA in modified human eutopic EECs by Western blot (up) and quantitation (down). C, The EMT level of RhoAoe and siRhoA cells assessed by the expression of E‐cadherin, vimentin and N‐cadherin (left) and quantitative analysis (right). D, The migration of control cells (up), RhoAoe cells (middle) and siRhoA cells (lower). E, Random ten visions of cell migration were quantified. F, The proliferation of control cells, RhoAoe cells and siRhoA cells explored by CCK‐8 assay within 120 h. G, EdU assays performed in control cells, RhoAoe cells and siRhoA cells (upper) and the quantitative analysis (lower). Data are expressed as mean ± SEM. *P < 0.05, **P < 0.001, ***P < 0.0001 vs control group

To assess the effect of RhoA/ROCK pathway in endometriosis, RhoA‐overexpression (RhoAoe) and RhoA‐knockdown (siRhoA) human eutopic EECs were constructed (Figure S1C). The RhoA was significantly increased in RhoAoe cells and that was enormously reduced in siRhoA cells (Figure 2A,B). As shown in Figure 2C, the enhancement of RhoA attenuated E‐cadherin and raised vimentin and N‐cadherin, and the reduction of RhoA exhibited opposite effect. Moreover, the migration of RhoAoe cells was remarkably promoted, and that of siRhoA cells was obviously decreased (Figure 2D,E). In addition, the results of Figure 2F indicated that RhoA obviously advanced the proliferation. And the augment of RhoA enhanced the EdU‐positive cells, which presented that RhoA up‐regulated the cell division to elevate the cell proliferation (Figure 2G). Therefore, the increasement of RhoA enhanced EMT, cell mobility and proliferation of human eutopic EECs, which reflected the effect of RhoA/ROCK pathway in endometriosis.

### 3.3 RhoA/ROCK pathway mediated the effect of oestrogen on EMT and proliferation

**FIGURE 3:**
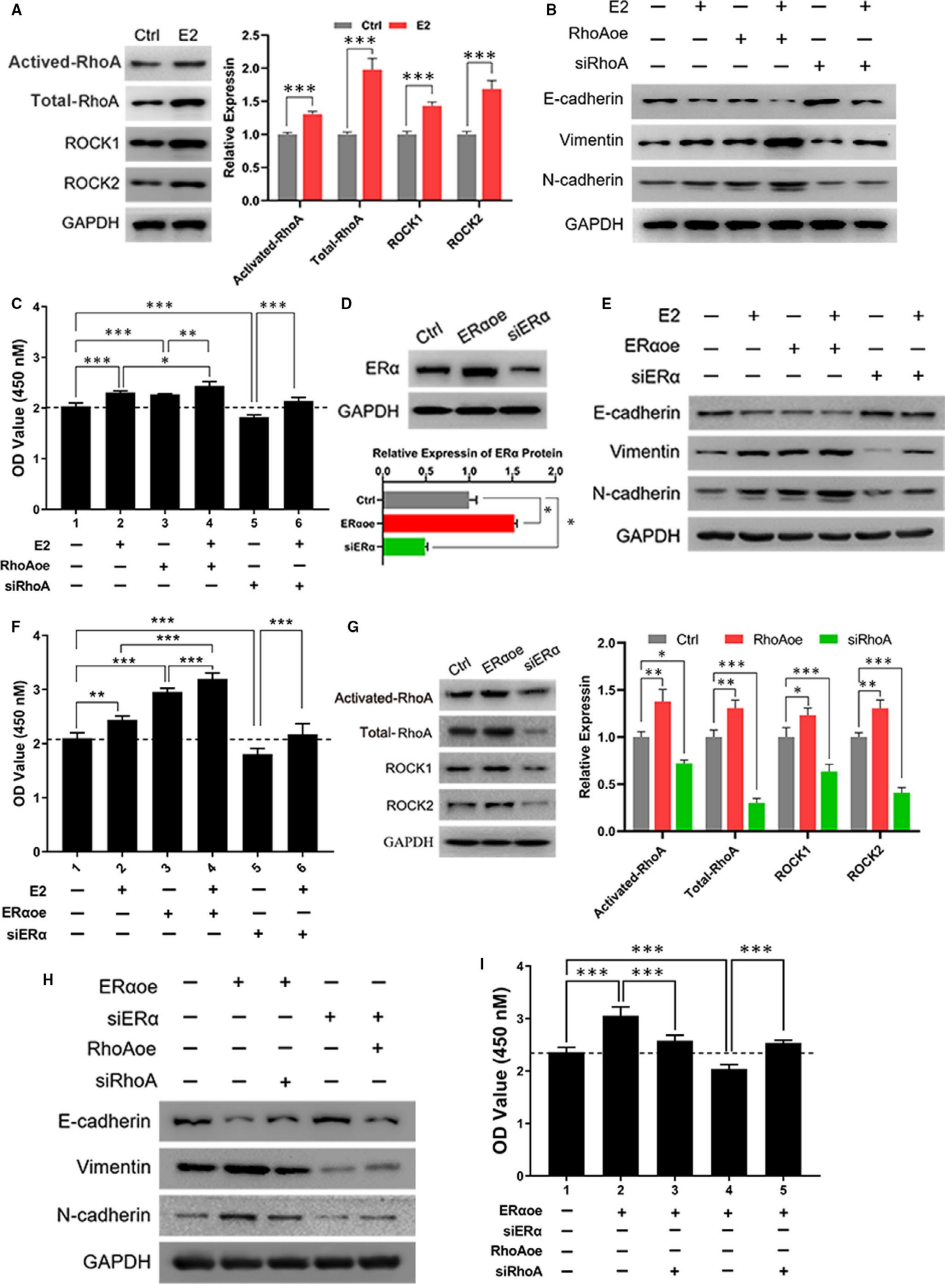
RhoA/ROCK pathway was responsible for the effect of oestrogen and ERα promoting EMT and proliferation. A, Oestrogen up‐regulated the RhoA activity and expression of RhoA, ROCK1 and ROCK2 (left) and quantitative analysis (right). B, The effect of oestrogen on EMT marker proteins was modulated by RhoA. C, The analysis of proliferation in oestrogen‐treated EECs or RhoA‐modified EECs. D, The expression of ERα in ERα overexpressed cells and ERα knockdown cells by Western blot (upper) and quantitation (lower). Ctrl: control cell, ERαoe: ERα‐overexpressed cells, siERα: ERα‐knockdown cells. E, The analysis of relationship between oestrogen and ERα on regulating EMT. F, The cell growth of E2‐treated cell, ERαoe cells, siERα cells or cotreated cells. G, The state of RhoA/ROCK pathway was changed in ERαoe and siERα cells without treatment of oestrogen by Western blot (left) and quantitative analysis (right). H, The effect of ERα on EMT was modified by RhoA. I, The examination of cell proliferation with the regulation of ERα and RhoA. Data are expressed as mean ± SEM. *P < 0.05, **P < 0.001, ***P < 0.0001 vs control group

Endometriosis is considered as an oestrogen‐dependent disorder, and we hypothesized that oestrogen might be an upstream regulator of RhoA/ROCK pathway. The results of Figure 3A implied that oestrogen remarkably promoted the transition of activated RhoA and the protein expression of RhoA and ROCK1/2. Further explorations found that the effect of oestrogen promoting EMT was markedly strengthened by enhancement of RhoA and lessened by loss of RhoA (Figure 3B and Figure S2A). As in Figure 3C, the effect of oestrogen regulating the proliferation of eutopic EECs was affected by the expression of RhoA. Consequently, these results suggested that RhoA/ROCK was responsible for the regulation of oestrogen on advancing EMT and proliferation in endometriosis.

### 3.4 RhoA/ROCK pathway was modulated by ERα, which was involved with regulation of oestrogenic function

ERα, as one of important oestrogen receptors, mediates the effect and function of oestrogen. To further analyse the function of ERα on oestrogen signalling in endometriosis, the ERα‐overexpression (ERαoe) and ERα‐knockdown (siERα) cells were established by lentivirus engineering (Figure S2B). And the expression of ERα was confirmed by Western blot (Figure 3D). As Figure 3E and Figure S2C indicated that overexpression of ERα up‐regulated the level of EMT and knockdown of ERα repressed the EMT of cells. Furthermore, the enrichment of ERα reinforced the function of oestrogen. Additionally, the increasement of ERα could further enhance the proliferation‐promoting effect of oestrogen, and loss of ERα impaired the function of oestrogen (Figure 3F), which illustrated that ERα might partially participate in mediating regulation of oestrogen signalling on EMT and proliferation.

Further investigations were employed to explore the effect of ERα on RhoA/ROCK pathway. As Figure 3G exhibited, the activity of RhoA and expression of RhoA, ROCK1 and ROCK2 were remarkably enhanced in ERαoe cells, and significantly decreased in siERα cells. The EMT‐promoting effect of ERα was potentiated by overexpression of RhoA, and repressed by reduction of RhoA (Figure 3H and Figure S2D). Moreover, modulating the expression of RhoA could influence the regulation of ERα on proliferation (Figure 3I). In conclusion, ERα might partially mediate the regulation of oestrogen and modulated RhoA/ROCK pathway to enhance the EMT level and proliferation.

### 3.5 ERK pathway was stimulated by oestrogen and ERα and promoted RhoA/ROCK pathway

**FIGURE 4:**
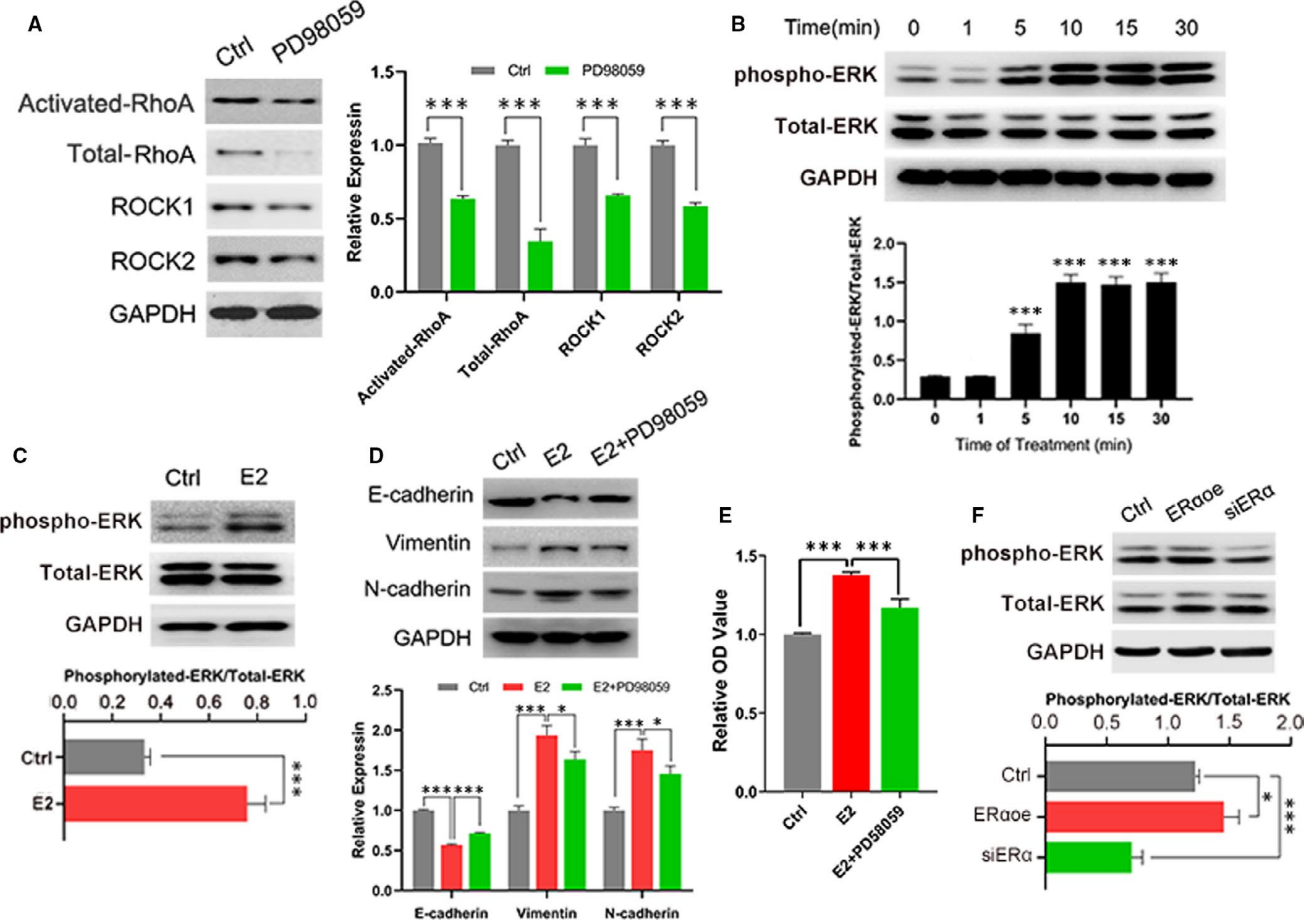
Oestrogen and ERα advanced the phosphorylation of ERK which modulated RhoA/ROCK pathway. A, Western blot detected the state of RhoA/ROCK pathway with influence of inhibitor of ERK (left) and quantitative analysis (right). PD98059, an inhibitor of ERK. B, Oestrogen promoted the expression of phosphorylated ERK in a times‐dependent way (upper) and quantitation (lower). The statistics analysis of groups was contrasted with the expression of 0 min. phospho‐ERK: phosphorylated ERK. C, Treatment of oestrogen for 48 h up‐regulated the expression of phosphorylated ERK (upper) and quantitative analysis (lower). D, The inhibition of ERK partially reversed the effect of oestrogen enhancing EMT (upper) and quantification (lower). E, The suppression of ERK depressed the function of oestrogen increasing the proliferation. F, ERα promoted the expression of phosphorylated ERK explored by Western blot (upper) and quantitative analysis (lower). Data are expressed as mean ± SEM. *P < 0.05, ***P < 0.0001 vs control group

Further researches were performed and found that inhibition of ERK signalling by PD98059 decreased the level of activated RhoA, total RhoA, ROCK1 and ROCK2 (Figure 4A), which implied ERK pathway might involve with the regulation of RhoA/ROCK pathway in endometriosis. The results of Figure 4B indicated that oestrogen increased the expression of phosphorylated ERK by the way of time dependence. And the treatment of 100 μmol/L oestrogen for 48 hours also obviously enhanced the level of phosphorylated ERK (Figure 4C). Moreover, the inhibitor of ERK, PD98059, distinguishably depressed the function of oestrogen on EMT and proliferation (Figure 4D,E). Further investigations found that the overexpression of ERα enhanced phosphorylated ERK and siERα cells contained less phosphorylated ERK (Figure 4F). Consequently, ERK signal might as a mediator of oestrogen/ERα regulating RhoA/ROCK pathway.

### 3.6 Oestrogen promoted the development of endometriosis in mouse model

**FIGURE 5:**
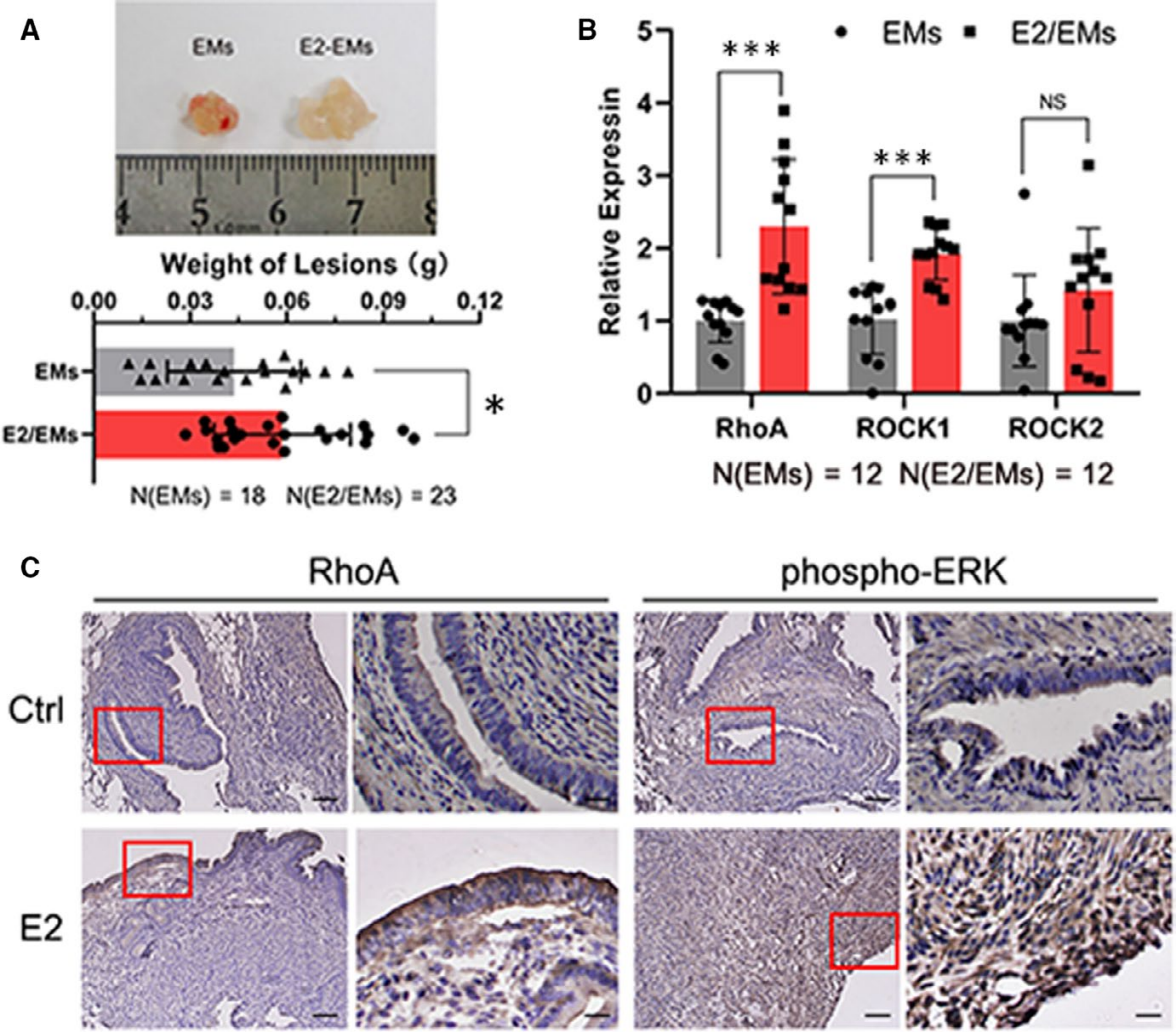
Oestrogen up‐regulated the development of endometriosis and expression of RhoA、ROCK1/2 and phosphorylated ERK in endometriotic mouse models. A, Consistent treatment of oestrogen enhanced the size (upper) and weight of mouse endometriotic lesions (lower). EM: PBS‐injected endometriosis group, E2/EM: E2‐injected endometriosis group. B, The mRNA expression of RhoA and ROCK1/2 was increased in the E2/EM group. C, Oestrogen increased the expression of RhoA and phosphorylated ERK in mouse endometriotic lesions. Data are expressed as mean ± SEM. NS = no significant difference, *P < 0.05, ***P < 0.0001 vs control group. Figure S1 (A) Quantitative analysis of Rho/ROCK pathway referred to GEO database. B, The mRNA expression of RhoB and RhoC from different human endometria. C, The images of cell morphology and green fluorescent protein (GFP) in RhoAoe and siRhoA cells detected by fluorescence microscope. Optical: optical microscope. Data are expressed as mean ± SEM. NS = no significant difference, *P < 0.05, **P < 0.001, ***P < 0.0001 vs control group

To verify the function of oestrogen and RhoA/ROCK pathway on the development of endometriosis in vivo, endometriosis mouse model was established. As shown in Figure 5A, the endometriotic lesions of the E2/EM group were much bigger than PBS‐injected EM group, and the weight of lesion from E2/EM group was much heavier than the control EM group. Furthermore, the expression of RhoA, ROCK1 and ROCK2 mRNA was enhanced in E2/EM endometriotic lesions (Figure 5B). And the RhoA and phosphorylated ERK were remarkably increased in E2‐treated group (Figure 5C). Thus, oestrogen promoted the development of endometriosis in mouse model and up‐regulated RhoA and phosphorylated ERK.

### 3.7 Discussions

In this research, we found that the protein expression of Rho/ROCK signalling pathway was abnormally changed in endometriosis and RhoA and ROCK1/2 were remarkably increased in eutopic and ectopic endometria. The mobility, EMT and proliferation of human eutopic EECs were promoted by enhancement of RhoA and depressed by knockdown of RhoA. Oestrogen enhanced the activity of RhoA and expression of RhoA, ROCK1 and ROCK2. Modulating RhoA expression resulted in the change of oestrogen effect on up‐regulating EMT and proliferation. ERα was involved with the function of oestrogen on enhancing the level of EMT and proliferation. Overexpression of ERα significantly up‐regulated RhoA/ROCK pathway, and ERα knockdown repressed it. Moreover, the effect of ERα on EMT and proliferation was also influenced by the change in RhoA expression. Oestrogen and ERα stimulated phosphorylation of ERK, which augmented the level of RhoA and ROCKs. Inhibition of ERK negatively affected the results of oestrogen on EMT and proliferation. Moreover, the endometriotic mouse model assay indicated that oestrogen enhanced the formation of endometriotic lesions. The expression of RhoA, ROCK1/2 and phosphorylated ERK was significantly elevated with the influence of oestrogen. To sum up, we conclude that RhoA/ROCK pathway mediated oestrogen/ERα/ERK signalling to promote the EMT and proliferation, resulting in further development of endometriosis.

Our previous research suggested that oestrogen that up‐regulated in endometriotic lesions enhanced the expression of IL‐6 and TNF‐α via p38 MAPK pathway resulting in inflammatory response to promote endometriosis.^23^ And oestrogen dramatically promoted EMT and MMP expression, which contributed the progression of endometriosis.^24^ Furthermore, it was reported that oestrogen promoted proliferation by inhibiting PTEN pathway, which may finally promote the proliferation of ectopic endometrial epithelial cells contributing to endometriosis.^25^ Oestrogen significantly promotes inflammation, EMT and proliferation, which aggressively promoted endometriosis, which involved with multiple signal pathway such as MAPK pathway. Thus, comprehensive and thorough study on the mechanism of oestrogen is instructive to the search for the treatment of endometriosis.

ERα, as one of the most important oestrogen receptors, mediates the function of oestrogen.^26^ It was reported that gonadotropin‐releasing hormone agonist (GnRH‐a) treatment especially decreased ERα mRNA levels in endometriotic cysts, which suggests reduction of ERα is an important step in the progression of endometriosis therapy by anti‐oestrogen treatment.^27^ Other research revealed that ERα gene polymorphism and recurrence of endometriosis were statistically significantly correlated. ^28^ In this study, we found ERα promoted the mobility, EMT and proliferation as a partial mediator of the oestrogen. However, the expression of ERα was decreased in endometriosis tissues comparing to eutopic and normal endometria in other reports.^29^, ^30^ And this interesting phenomenon highlights the complexity and particularity of endometriosis that is not as extreme like malignant tumours. Briefly, ERα partially mediates the effect of oestrogen promoting EMT and proliferation in endometriosis.

It is reported that the level of phosphorylated ERK was significantly higher in endometriotic lesions than in endometrial tissues.^31^, ^32^ And the activated ERK regulated over 160 proteins, which involved with the cell proliferation, apoptosis, angiogenesis and migration in endometriosis.^33^, ^34^, ^35^ As exhibited in previous results, oestrogen could trigger phosphorylation of ERK within a pretty short time, and treatment of a long time also up‐regulated the level of phosphorylated ERK. Thus, the effect of oestrogen on ERK signalling might be through multiple ways. And inhibition of ERK partly reduced the promoting oestrogenic effect on EMT and cell growth, which implied ERK signal played a limited role on oestrogen pathway. On the other hand, ERα also could regulate the expression of phosphorylation of ERK, suggesting that ERα might serve as a mediator of oestrogen to manage ERK signal.

Rho/ROCK pathway is involved in the regulation of cytoskeletal proteins, cell morphology, migration, and various proliferation and transcriptional activities in cells.^22^, ^36^ Thus, the dysfunction of Rho/ROCK pathway in endometriosis confirmed by previous data might dramatically modulate internal structure and fate of cells. Research showed that oestrogen regulates motility of human epididymal smooth muscle cells by increasing RhoA/ROCK signalling.^37^ And Oviedo et al disclosed estradiol enhances the RhoA/ROCK pathway and increases cell cycle–related protein expression by acting through oestrogen receptors.^38^ Another study found that ERα enhanced ROCK2/MMP2/9 pathway inducing cell migration and angiogenesis effects in endothelial cell.^39^ These findings suggested that there may be involved many pathways in the process of oestrogen regulating RhoA/ROCK pathway. The expression of RhoA and ROCK1/2 was up‐regulated by oestrogen, ERα and ERK. Furthermore, the activity of RhoA was increased accompanied by the enhancement of total RhoA, which implied that increasement of total RhoA might induce the activation of RhoA. However, whether the detailed mechanism of RhoA activation was involved with other factors needs further study. Consequently, our present study concluded that oestrogen/ERα/ERK signal up‐regulated the activation of RhoA and expression of RhoA and ROCK1/2 triggering the enhancement of EMT and proliferation.

In conclusion, this study specifically revealed RhoA/ROCK pathway was up‐regulated by oestrogen/ERα/ERK signalling pathway promoting EMT and proliferation in endometriosis. Thus, RhoA/ROCK pathway might be an attractive molecular target in anti‐oestrogen treatment of endometriosis. These results provide a scientific basis for potential therapeutic strategies to treat endometriosis via RhoA/ROCK pathway.

## CONFLICT OF INTERESTS

The author(s) announced there were no potential conflicts of interest in this article.

## AUTHOR CONTRIBUTION

**Zhi‐Xiong Huang:** Data curation (lead); Investigation (lead); Software (lead); Writing‐original draft (lead). **Xiao‐Mei Mao:** Data curation (equal); Investigation (equal); Writing‐original draft (equal). **Rong‐Feng Wu:** Investigation (supporting); Resources (supporting); Validation (supporting). **Shao‐Min Huang:** Formal analysis (equal); Funding acquisition (supporting); Investigation (equal); Resources (supporting). **Xin‐Yu Ding:** Software (equal); Validation (equal). **Qing‐Xi Chen:** Funding acquisition (equal); Writing‐review & editing (equal). **Qiong‐Hua Chen:** Conceptualization (lead); Funding acquisition (lead); Methodology (lead); Project administration (lead); Writing‐review & editing (lead).

## Supporting information

Fig S1

Fig S2

Table S1

## Data Availability

All data included in this study are available upon request by contact with the corresponding author.
