# Supplementary figures and images for: RhoA/ROCK pathway mediates the effect of oestrogen on regulating epithelial‐mesenchymal transition and proliferation in endometriosis
Source: J Cell Mol Med. 2020 Jul 29;24(18):10693–704. doi: 10.1111/jcmm.15689 (PMC7521234; doi:10.1111/jcmm.15689)

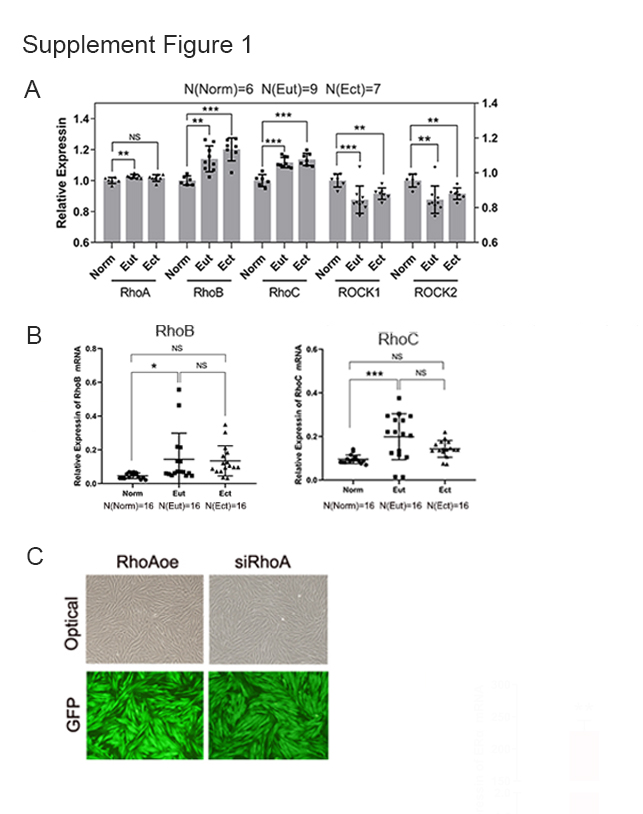

Supplement: Supplementary file 1 — Fig S1 [file JCMM-24-10693-s001.jpg]

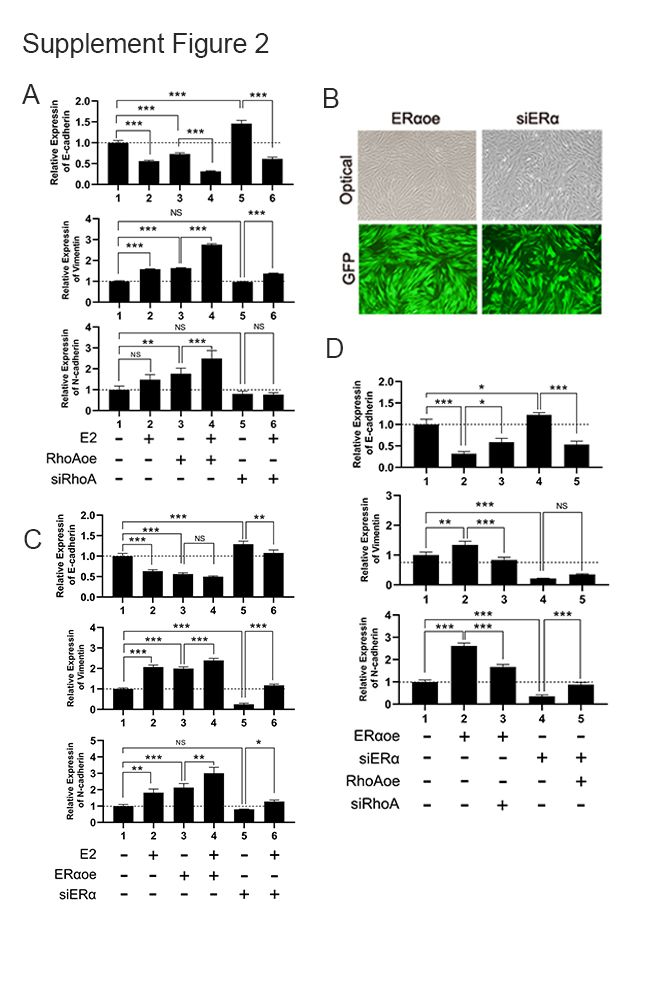

Supplement: Supplementary file 2 — Fig S2 [file JCMM-24-10693-s002.jpg]
