# Supplementary material for: RhoA/ROCK pathway mediates the effect of oestrogen on regulating epithelial‐mesenchymal transition and proliferation in endometriosis
Source: J Cell Mol Med. 2020 Jul 29;24(18):10693–704. doi: 10.1111/jcmm.15689 (PMC7521234; doi:10.1111/jcmm.15689)
Supplement: Supplementary file 3 — Table S1 [file JCMM-24-10693-s003.docx]

| Gene Name | Sequences (5’-3’) |
| --- | --- |
| Human-GAPDH | Forward: GGAAGGTGAAGGTCGGAGTCA  Reverse: GAGTCCTTCCACGATACCAA |
| Human-RhoA | Forward: AGCCTGTGGAAAGACATGCTT  Reverse: TCAAACACTGTGGGCACATAC |
| Human-RhoB | Forward: CTGCTGATCGTGTTCAGTAAGG  Reverse: TCAATGTCGGCCACATAGTTC |
| Human-RhoC | Forward: GGAGGTCTACGTCCCTACTGT  Reverse: CGCAGTCGATCATAGTCTTCC |
| Human-ROCK1 | Forward: AACATGCTGCTGGATAAATCTGG  Reverse: TGTATCACATCGTACCATGCCT |
| Human-ROCK2 | Forward: TCAGAGGTCTACAGATGAAGGC  Reverse: CCAGGGGCTATTGGCAAAGG |
| Human-ERα | Forward: CCACCAACCAGTGCACCATT  Reverse: GGTCTTTTCGTATCCCACCTTTC |
| Mouse-GAPDH | Forward: TGACCTCAACTACATGGTCTACA  Reverse: CTTCCCATTCTCGGCCTTG |
| Mouse-RhoA | Forward: AGCTTGTGGTAAGACATGCTTG  Reverse: GTGTCCCATAAAGCCAACTCTAC |
| Mouse-ROCK1 | Forward: GACTGGGGACAGTTTTGAGAC  Reverse: ATCCAAATCATAAACCAGGGCAT |
| Mouse-ROCK2 | Forward: GGTTTACAGATGAAAGCGGAAGA  Reverse: GTGATGCCTTATGACGAACCAA |

**Supplement Table** Primer sequences used in qRT-PCR analysis.
